# Single-Cell Approach to Influenza-Specific CD8^+^ T Cell Receptor Repertoires Across Different Age Groups, Tissues, and Following Influenza Virus Infection

**DOI:** 10.3389/fimmu.2018.01453

**Published:** 2018-06-27

**Authors:** Sneha Sant, Ludivine Grzelak, Zhongfang Wang, Angela Pizzolla, Marios Koutsakos, Jane Crowe, Thomas Loudovaris, Stuart I. Mannering, Glen P. Westall, Linda M. Wakim, Jamie Rossjohn, Stephanie Gras, Michael Richards, Jianqing Xu, Paul G. Thomas, Liyen Loh, Thi H. O. Nguyen, Katherine Kedzierska

**Affiliations:** ^1^Department of Microbiology and Immunology, Peter Doherty Institute for Infection and Immunity, University of Melbourne, Melbourne, VIC, Australia; ^2^École Normale Supérieure Paris-Saclay, Université Paris-Saclay, Cachan, France; ^3^Deepdene Surgery, Deepdene, VIC, Australia; ^4^Immunology and Diabetes Unit, St. Vincent’s Institute of Medical Research, Fitzroy, VIC, Australia; ^5^Lung Transplant Unit, Alfred Hospital, Melbourne, VIC, Australia; ^6^Infection and Immunity Program, Department of Biochemistry and Molecular Biology, Biomedicine Discovery Institute, Monash University, Clayton, VIC, Australia; ^7^ARC Centre of Excellence in Advanced Molecular Imaging, Monash University, Clayton, VIC, Australia; ^8^School of Medicine, Institute of Infection and Immunity, Cardiff University, Cardiff, United Kingdom; ^9^Victorian Infectious Diseases Service, The Royal Melbourne Hospital, Peter Doherty Institute for Infection and Immunity, Parkville, VIC, Australia; ^10^Shanghai Public Health Clinical Center, Institutes of Biomedical Sciences, Key Laboratory of Medical Molecular Virology of Ministry of Education/Health, Shanghai Medical College, Fudan University, Shanghai, China; ^11^Department of Immunology, St Jude Children’s Research Hospital, Memphis, TN, United States

**Keywords:** T cell receptor repertoire, influenza A virus, human T lymphocytes, CD8^+^ T cells, aging, tissues

## Abstract

CD8^+^ T cells recognizing antigenic peptides derived from conserved internal viral proteins confer broad protection against distinct influenza viruses. As memory CD8^+^ T cells change throughout the human lifetime and across tissue compartments, we investigated how T cell receptor (TCR) composition and diversity relate to memory CD8^+^ T cells across anatomical sites and immunological phases of human life. We used *ex vivo* peptide-HLA tetramer magnetic enrichment, single-cell multiplex RT-PCR for both the TCR-alpha (TCRα) and TCR-beta (TCRβ) chains, and new TCRdist and grouping of lymphocyte interactions by paratope hotspots (GLIPH) algorithms to compare TCRs directed against the most prominent human influenza epitope, HLA-A*02:01-M1_58–66_ (A2^+^M1_58_). We dissected memory TCR repertoires directed toward A2^+^M1_58_ CD8^+^ T cells within human tissues and compared them to human peripheral blood of young and elderly adults. Furthermore, we compared these memory CD8^+^ T cell repertoires to A2^+^M1_58_ CD8^+^ TCRs during acute influenza disease in patients hospitalized with avian A/H7N9 virus. Our study provides the first *ex vivo* comparative analysis of paired antigen-specific TCR-α/β clonotypes across different tissues and peripheral blood across different age groups. We show that human A2^+^M1_58_ CD8^+^ T cells can be readily detected in human lungs, spleens, and lymph nodes, and that tissue A2^+^M1_58_ TCRαβ repertoires reflect A2^+^M1_58_ TCRαβ clonotypes derived from peripheral blood in healthy adults and influenza-infected patients. A2^+^M1_58_ TCRαβ repertoires displayed distinct features only in elderly adults, with large private TCRαβ clonotypes replacing the prominent and public TRBV19/TRAV27 TCRs. Our study provides novel findings on influenza-specific TCRαβ repertoires within human tissues, raises the question of how we can prevent the loss of optimal TCRαβ signatures with aging, and provides important insights into the rational design of T cell-mediated vaccines and immunotherapies.

## Introduction

Influenza infections cause significant morbidity and mortality, with an estimated 3–5 million cases of severe illness and approximately 290,000–650,000 deaths worldwide annually^1^ (January 2018). In particular, young children, pregnant women, and elderly individuals are at high risk of severe influenza disease, often leading to high hospitalization rates. While current vaccination regimens, eliciting B cell and follicular T cell responses (1), are our most effective way of controlling annual influenza epidemics, the constantly changing nature of influenza viruses can easily evade preexisting host antibody responses (2). By contrast, killer CD8^+^ T cells are effective in inducing long-lived heterologous immunity toward different influenza strains (3, 4), thus leading to a rapid recovery of the host (5–7). CD8^+^ T cells eliminate influenza virus infection by recognizing conserved viral proteins presented by major histocompatibility complex (MHC) class I glycoproteins (8), thus protecting influenza-infected individuals against a diverse range of human-derived (i.e., H1N1 and H3N2), swine-derived (i.e., pandemic H1N1), and the more recently described deadly avian-derived (i.e., H5N1, H7N9, and H9N2) influenza viruses (9–11). Such recognition of peptide-MHC-I (p-MHC-I) complexes occurs *via* surface-expressed T cell receptors (TCRs) comprising of α and β chains. Following T cell activation, antigen-specific CD8^+^ T cells proliferate, migrate to the site of infection, and mount an immune response by killing virus-infected cells and producing anti-viral cytokines (12).

CD8^+^ T cells are highly specific in their interactions with p-MHC-I complexes (13, 14), and small changes within viral peptides can abrogate TCRαβ recognition, leading to viral escape from preexisting immunity (15–19). The vast array of different TCRs is generated by somatic recombination of variable (TRAV for TCRα chain and TRBV for TCRβ chain), diversity (within the β-chain only; Dβ), and joining (Jα and Jβ) gene segments in combination with varying lengths of the complementarity-determining region 3 (CDR3; CDR3α and CDR3β) loops. A set of different TCRαβ receptors, referred to as the TCRαβ repertoire, can recognize specific antigenic peptides in the context of MHC-I. TCRs exhibiting the same gene segment usage and amino acid (aa) sequences at CDR3α and CDR3β regions are defined as TCR clonotypes, and a range of TCR clonotypes displaying either near-identical or diverse features can recognize a given T cell epitope (20). Furthermore, TCR repertoires can be classified as “public,” when they express identical epitope-specific TCR sequences across multiple individuals (21–23), or “private,” when different individuals display distinct (non-overlapping) TCR clonotypes for the same p-MHC-I complex (21, 22, 24). The presence of “public” TCRαβ clonotypes is thought to have a selective evolutionary advantage, as these TCRs can be easily generated in different individuals (25–30).

As TCRs can greatly affect viral clearance, p-MHC-I avidity, and prevention from viral escape (31–33), it is important to understand TCR repertoire diversity and composition within influenza-specific CD8^+^ T cells directed at prominent human epitopes for designing a much-needed universal T cell-based influenza vaccine. To date, our knowledge of human influenza-specific CD8^+^ TCRs has been mainly focused on the HLA-A*02:01-restricted immunodominant M1_58–66_ (A2^+^M1_58_) epitope (7, 34–42), as HLA-A*02:01 is the most common HLA class I allele expressed at 1–54.5% (43) across different ethnic groups worldwide. For example, HLA-A*02:01 is expressed at 0.5–31% in Caucasians and 9–40% in native North Americans (42). Until recently (44), TCR analysis was mainly performed on bulk cells for a single TCRα or TCRβ chain, or following extensive CD8^+^ T cell cloning (45, 46). However, our recent studies using a single-cell multiplex RT-PCR approach (44) has showed, for the first time, direct *ex vivo* dissection of paired TCRαβ repertoires directed against the most prominent influenza A2^+^M1_58_ epitope in healthy adults (36), elderly individuals (40), as well as in hospitalized influenza-infected elderly patients (7). In general, the A2^+^M1_58_ TCRαβ repertoires were heavily biased toward TRBV19 and TRAV27, with public TRBV19/TRAV27-utilizing clonotypes being its main feature (34, 36), along with other less frequent diverse TCRs (37). Public TRBV19/TRAV27 motifs bearing CDR3α-GGSQGNL and CDR3β-SSIRSYEQ sequences are important for the optimal recognition of A2^+^M1_58_ epitope, especially the CDR3β-IRS motif being key for the peg-notch mode of recognition (23, 36, 37). However, it is far from clear how the A2^+^M1_58_ TCRαβ repertoire changes across different age groups, between different tissue compartments and following influenza virus infection. Especially, the composition of A2^+^M1_58_ TCRαβ repertoire is largely unknown in human tissues, as previous studies have mainly focused on human peripheral blood (9, 10, 35, 36, 47, 48), with only one recent report from our group focusing on different subsets of influenza-specific CD8^+^ T cells in human lungs (41).

Here, we dissected A2^+^M1_58_ TCRαβ repertoires across human tissues [lungs, spleen, and lymph node (LN)] and subsequently performed a comprehensive analysis of temporal dynamics of A2^+^M1_58_ TCRs across different age groups (young/elderly), in the context of acute influenza infection (H7N9-infected hospitalized elderly patients), and across different anatomical locations. Using a combination of direct *ex vivo* tetramer enrichment, single-cell paired TCRαβ analyses and the newly established algorithm TCRdist (38) and grouping of lymphocyte interactions by paratope hotspots (GLIPH) (39), we compared and contrasted A2^+^M1_58_ TCRαβ repertoires across the above-mentioned datasets. We show that human A2^+^M1_58_ CD8^+^ T cells can be readily detected in human lungs, spleens, and LNs, and that the tissue A2^+^M1_58_ TCRαβ repertoires reflect those from unmatched peripheral blood A2^+^M1_58_ TCRαβ repertoires in healthy adults and hospitalized influenza-infected patients. Strikingly, elderly individuals displayed unique features within their A2^+^M1_58_ TCRαβ repertoires, with largely expanded private TCRαβ clonotypes replacing, at least in part, the public TRBV19/TRAV27 TCRs. Our study provides novel findings on influenza-specific TCRαβ repertoires within human tissues and raises the question of how we can prevent the loss of optimal TCRαβ signatures with aging.

## Materials and Methods

### Human Peripheral Blood Samples

This study was approved by the University of Melbourne Human Ethics Committee (ID 1443389.3 and 0931311.5). Human experimental work was conducted according to the Declaration of Helsinki Principles and according to the Australian National Health and Medical Research Council (NHMRC) Code of Practice. All donors provided informed written consent for blood donation. Tissues from deceased organ donors were obtained following written informed consent from the next of kin. PMBCs were isolated from heparinized peripheral blood collected from healthy adult volunteers (AD, 22–59 years) at the University of Melbourne and elderly donors (ED, ≥60 years) recruited at Deepdene Medical Clinic (Deepdene, Australia), or from buffy packs of healthy adult donors (AD, 22–59 years) obtained from Australian Red Cross Blood Service (ARCBS). Peripheral blood samples from HLA-A*02:01^+^ H7N9-infected patients (A9, A10, and A79) were obtained from Shanghai Public Health Clinic Centre (SHAPHC) under the supervision of SHAPHC Ethics Committee (7). PBMCs were also obtained from one patient hospitalized at the Royal Melbourne Hospital in 2017 with seasonal-influenza virus infection and approved by the Monash Health Human Research Ethics Committee (HREC/15/MonH/64).

### Human Tissue Samples

Human spleens, LNs (mesenteric and pancreatic), and lung tissue samples were obtained from deceased organ donors following family’s approval and approved by the Australian Red Cross Blood Service Ethics Committee (ID 2015#8). Spleen (SP) and mesenteric/pancreatic LN samples were procured from DonateLife Victoria. Lung (LG) tissue was obtained from the Alfred Hospital’s Lung Tissue Biobank (supported by the NHMRC Lung Fibrosis CRE). All tissue samples were stored and transported in cold PBS and processed within 18 h of procurement. Spleen and LNs were finely chopped and then enzymatically digested with type III Collagenase (1 mg/ml, Worthington, OH, USA) and DNase I (0.5 mg/ml, Roche, Basel, Switzerland) in RPMI media for 1 h at 37°C/5% CO_2_ (45 min for LN). Enzymatic digestion was halted by the addition of EDTA (0.01 mM) before cells were passed through a 70 µm sieve, washed, and then red blood cells were lysed with RBC lysis solution (0.168 M ammonium chloride, 0.01 mM EDTA, and 12 mM sodium bicarbonate in MilliQ water) before cryopreservation. Lung tissues were similarly processed, with the exception of 7 mg/ml type III Collagenase and 1 mg/ml DNase I digestion, as previously described (41).

### *Ex Vivo* Detection of Influenza-Specific CD8^+^ T Cells

Between 2 × 10^7^ and 5 × 10^7^ of cells were thawed and washed in RPMI containing DNase I (1 mg/ml). Mouse splenic red pulp has been shown to contaminate magnetic column enrichments (49) and was also observed in our human spleen cell suspensions. Thus, to circumvent the contamination of our tetramer enrichments, spleen cells were thawed and passed through an LS column (Miltenyi Biotec, Bergisch Gladbach, Germany) before use. Blood and tissue cell preparations underwent tetramer-associated magnetic enrichment (TAME) to enrich for A2^+^M1_58_-specific CD8^+^ T cells using the A2^+^M1_58_-tetramer, essentially as previously described (40). Cell fractions (except for lung cells) were surface stained with anti-CD3-PECF594 (clone: UCHT1, BD, San Jose, CA, USA), anti-CD8-PerCpCy5.5 (clone: SK1, BD Pharmingen), anti-CD14-APCH7 (clone: MFP9, BD Pharmingen), anti-CD19-APCH7 (clone: SJ25C1, BD Pharmingen), anti-CD27-BV711 (clone: L128, BD Horizon), anti-CD45RA-FITC (clone: HI100, BD Pharmingen), and LIVE/DEAD™ Fixable Near-IR (Molecular Probes, Eugene, OR, USA). Lymphocytes isolated from lung tissue (41) and H7N9-infected patients (7) did not undergo TAME enrichment, instead, these samples were stained directly *ex vivo* with A2^+^M1_58_-tetramer for 1 h at room temperature, washed and stained with surface markers, as previously described. Lung cells were surface stained as previously described (41) with anti-CD103-FITC (clone: Ber-ACT8, Biolegend), anti-CD45RO-PECy7 (clone: UCHL1, eBioscience), anti-CD69-BV421 (clone: FN50, Biolegend), anti-CD8-APCH7 (clone: SK1, Biolegend) and anti-CD3-APC (clone: OKT3, eBioscience). Splenic cells and PBMCs were also surface stained as above to compare the frequency of resident-T cells with lung, spleen and blood. Cells were resuspended in PBS containing 0.5% BSA/2 mM EDTA for single-cell sorting on a FACSAria III (BD Biosciences) or fixed with 1% PFA and acquired on an LSRFortessa II (BD Biosciences). FACS files were analyzed using FlowJo software (V10.2, Treestar, Ashland, OR, USA).

### Single-Cell Paired TCRαβ Analysis

Following TAME or direct A2^+^M1_58_-tetramer staining, single live CD3^+^CD8^+^tetramer^+^CD19^−^CD14^−^ cells were sorted into pre-chilled 96-well twin.tec plates (Eppendorf, Hamburg, Germany) before performing multiplex-nested RT-PCR for paired TCRαβ analysis essentially as described (36, 40, 41, 44). TCRα and TCRβ sequences were parsed using the TCRdist algorithm (38). CDR3αβ pairs displaying the same aa sequences and V/J gene usage were defined as clonotypes. Heatmaps for CDR3α/β were made using Matplotlib (50). Circos plots were generated using Circos package (51).

### Statistical Analysis

Statistical analysis was carried out using GraphPad prism software. Student’s *t*-test was performed for comparison between two groups. Statistical significance if present is described in figure legend.

### TCRβ Cluster Analysis

CDR3β aa sequences obtained after parsing through TCRdist were used to construct clone network analysis using GLIPH (39). Based on the presence of unique motifs in a given dataset, GLIPH was used to calculate the probability of the occurrence of these unique motifs relative to their expected frequency in a naïve TCR dataset. The analysis returned a network of all CDR3β’s as nodes and global, local or singleton interactions as edges. Network clusters were generated using the clusterMaker plugin in Cytoscape V3.5.1.^2^

### Datasets Used in this Study

Comprehensive analyses of A2^+^M1_58_^+^CD8^+^ TCRαβ repertoire diversity was analyzed using paired TCRαβ datasets from donor spleens (SP234, SP235, SP156, and SP583), LNs (LN583), and lungs (LG9 and LG10) generated in this study, as well as from our previously published datasets from peripheral blood TCRαβ clonotypes found in adults (*n* = 3, AD9, AD16, and AD17) (36), elderly (*n* = 3, ED9, ED18, and ED31) (40), H7N9-infected patients (*n* = 3, A9, A10, and A79) (7), and one lung tissue [LG6 (41)]. Therefore, our total dataset (Table 1) comprised of 628 paired TCRαβ sequences that was parsed through TCRdist algorithm, analyzed using GLIPH, and visualized using Cytoscape to contribute to Figures 3–8. Sequence data from our study and the published studies have been made publicly available at VDJDb (52).

**Table 1:** Demographics of donors used for T cell receptor (TCR) analyses in this study.

Group	Donor	Age (years)	Total no. of TCRs	No. of TCRs from individual donors	Figure
Adult	AD 16 (36)	46	85	26	Figures 3–8
	AD 17 (36)	29		26	
	AD 19 (36)	28		33	

Elderly	ED 9 (40)	76	86	28	Figures 3–8
	ED 31 (40)	61		38	
	ED 18 (40)	66		20	

H7N9 patients	A 9 (7)	67	195	39	Figures 3–8
	A 10 (7)	65		76	
	A 79 (7)	78		80	

Lung	LG 10	42	129	25	Figures 2–8
	LG 9 (41)	28		35	
	LG 6	50		69	

Spleen	SP 583	38	108	13	Figures 2–8
	SP 156	69		36	
	SP 235	57		31	
	SP 234	48		28	
Lymph node	LN 583	38	25	25	

## Results

### A2^+^M1_58_^+^CD8^+^ T Cells Are Maintained Across Age, Tissues, and During Acute Influenza Infection

**Figure 1:**
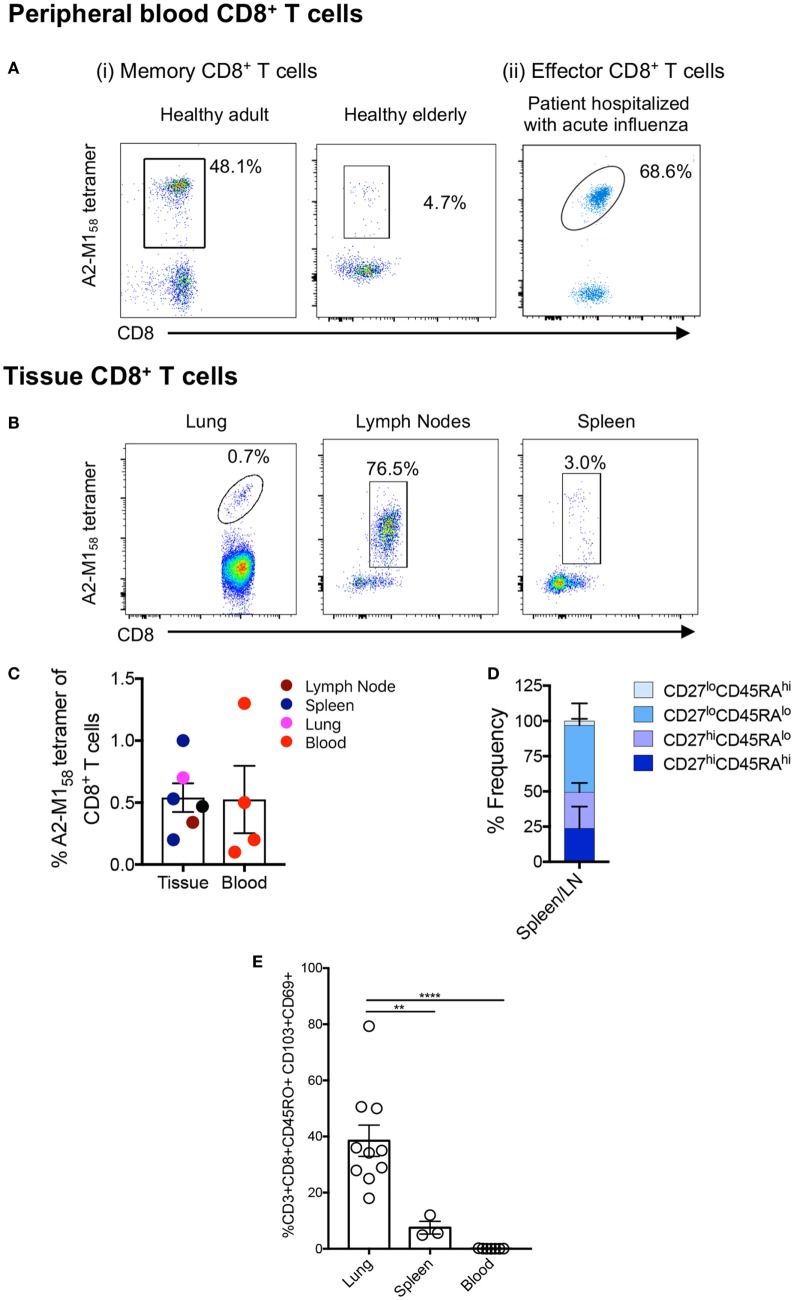
A2^+^M1_58_-specific CD8^+^ T cells are prominent across age, tissue, and influenza infection. Representative FACS plots of A2^+^M1_58_-specific memory CD8^+^ T cells in the blood of [**(A)** i] healthy adult and an elderly donor, [**(A)** ii] in the blood of influenza virus-infected hospitalized patient, and **(B)** in the lung, spleen, and lymph nodes (LNs) of deceased organ donors. Samples were enriched *via* tetramer-associated magnetic enrichment (TAME), except for the lung which was directly stained with tetramer. Frequencies of cells gated as viable CD14^−^CD19^−^CD3^+^CD8^+^ A2^+^M1_58_-tetramer-positive events are shown. **(C)** Frequencies of A2/M1_58_^+^CD8^+^ T cells in tissues (LNs *n* = 1 donor, spleen *n* = 4 donors, and lung *n* = 1 donor) and blood (*n* = 3 donors) for A2/M1_58_^+^CD8^+^ T cells are shown. Except for the lung, which did not undergo TAME enrichment, A2^+^M1_58_-tetramer^+^CD8^+^ T cell frequencies of total CD8^+^ T cells were calculated based on the total CD8^+^ T cell population. No statistical differences across distinct anatomical compartments were found. **(D)** CD45RA/CD27 memory profiles of A2^+^M1_58_-specific CD8^+^ T cells within human tissues are shown. **(E)** Graph depicts the proportion of CD8^+^ TRMs (CD103^+^CD69^+^) among the total memory CD8^+^ T cell pool in the lung, spleen, and blood of donors (41). Dots represent individual donors (*n* = 3–10 donors, one-way ANOVA, Sidak’s multiple comparison).

Effective CD8^+^ T cell responses are governed by several factors, including available numbers of naïve or memory CD8^+^ T cells against the pathogen, as well as the maintenance of antigen-experienced memory CD8^+^ T cells with aging and across tissue compartments (53–55). Using A2^+^M1_58_-TAME (56, 57), A2^+^M1_58_-specific CD8^+^ T cells can be readily detected in human peripheral blood of healthy adult and elderly individuals (Figure 1Ai) as well as human spleens (*n* = 3) and LNs (*n* = 1), and in unenriched human lungs (*n* = 3) (Figure 1B; Table 1). This clearly demonstrates that distribution of human influenza-specific A2^+^M1_58_^+^CD8^+^ T cells across different anatomical sites is analogous to that of influenza-specific immunodominant D^b^PA_224_^+^CD8^+^ (24) and D^b^NP_366_^+^CD8^+^ (21) T cells in mice. Furthermore, effector memory A2^+^M1_58_^+^CD8^+^ T cells can be detected directly *ex vivo* in hospitalized patients infected with influenza viruses *via* tetramer enrichment (Figure 1Aii). Thus, A2^+^M1_58_^+^CD8^+^ T cells are prominent in HLA-A*02:01-expressing individuals of different ages across different tissue compartments, with comparable tetramer frequencies of total CD8^+^ T cells found between blood and tissues (Figure 1C).

We have previously characterized the memory phenotypes of A2^+^M1_58_^+^CD8^+^ T cells from the blood of adult and elderly adult donors, which mainly expressed CD27^hi^, but not CD45RA (40), a marker highly expressed in naïve T cells. By contrast, A2^+^M1_58_^+^CD8^+^ T cells in H7N9-infected patients displayed a more effector-like phenotype (CD38^hi^HLA-DR^hi^CD27^lo^CD45RA^lo^) (7). In tissues, the A2^+^M1_58_^+^CD8^+^ T cells were of a CD27^hi/lo^CD45RA^lo^ memory phenotype in spleens and LNs (Figure 1D), whereas in the lung, they were positive for the CD45RO^+^ memory marker and displayed a mixture of circulating (CD69^−^CD103^−^ and CD69^+^CD103^−^) and tissue-resident (CD69^+^CD103^+^) memory phenotypes (41). Human spleens contained significantly less CD69^+^CD103^+^ tissue-resident memory (TRM) T cells compared to the lung, with relatively few TRMs observed in the blood (Figure 1E).

### TCRαβ Repertoire of A2^+^M1_58_^+^CD8^+^ T Cells in Human Tissues

**Figure 2:**
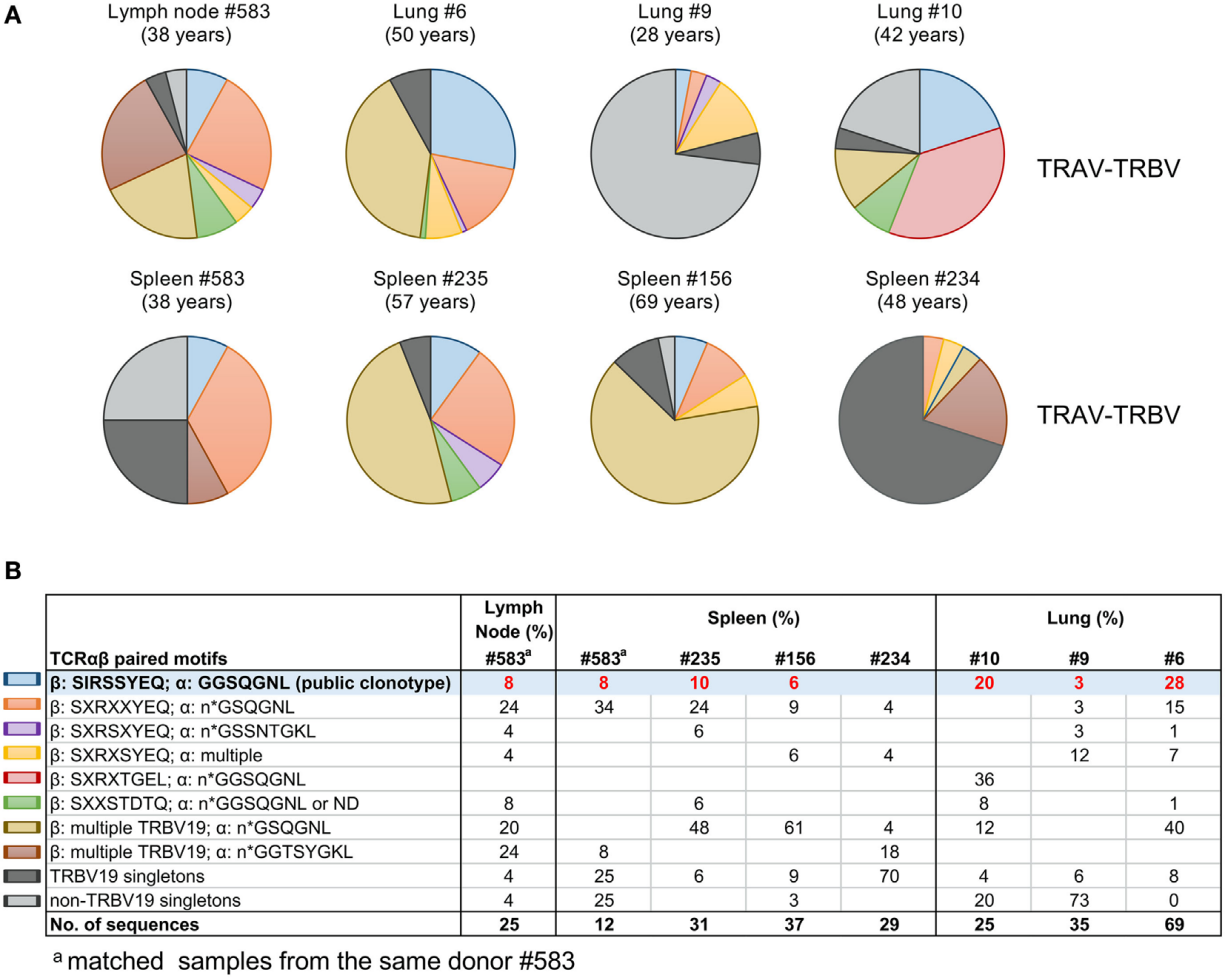
Tissue A2^+^M1_58_-specific CD8^+^ T cell receptors (TCRs) display public clonotype signatures. **(A)** Pie-chart distributions and **(B)** table of paired TCRα/β clonotype repertoires of A2^+^M1_58_-specific memory CD8^+^ T cells in human tissues. TRB-TRA clonotypes were grouped according to the presence of “public” [blue pie-slice in **(A)** and shaded in **(B)**] or public-like motifs (other colors). TRBV19 singletons are in dark gray and non-TRBV19 singletons in light gray. Singletons are defined as TCRβ chain clonotypes with variable TCRα chains. Matched samples from the same donor #583 are indicated.

**Figure 3:**
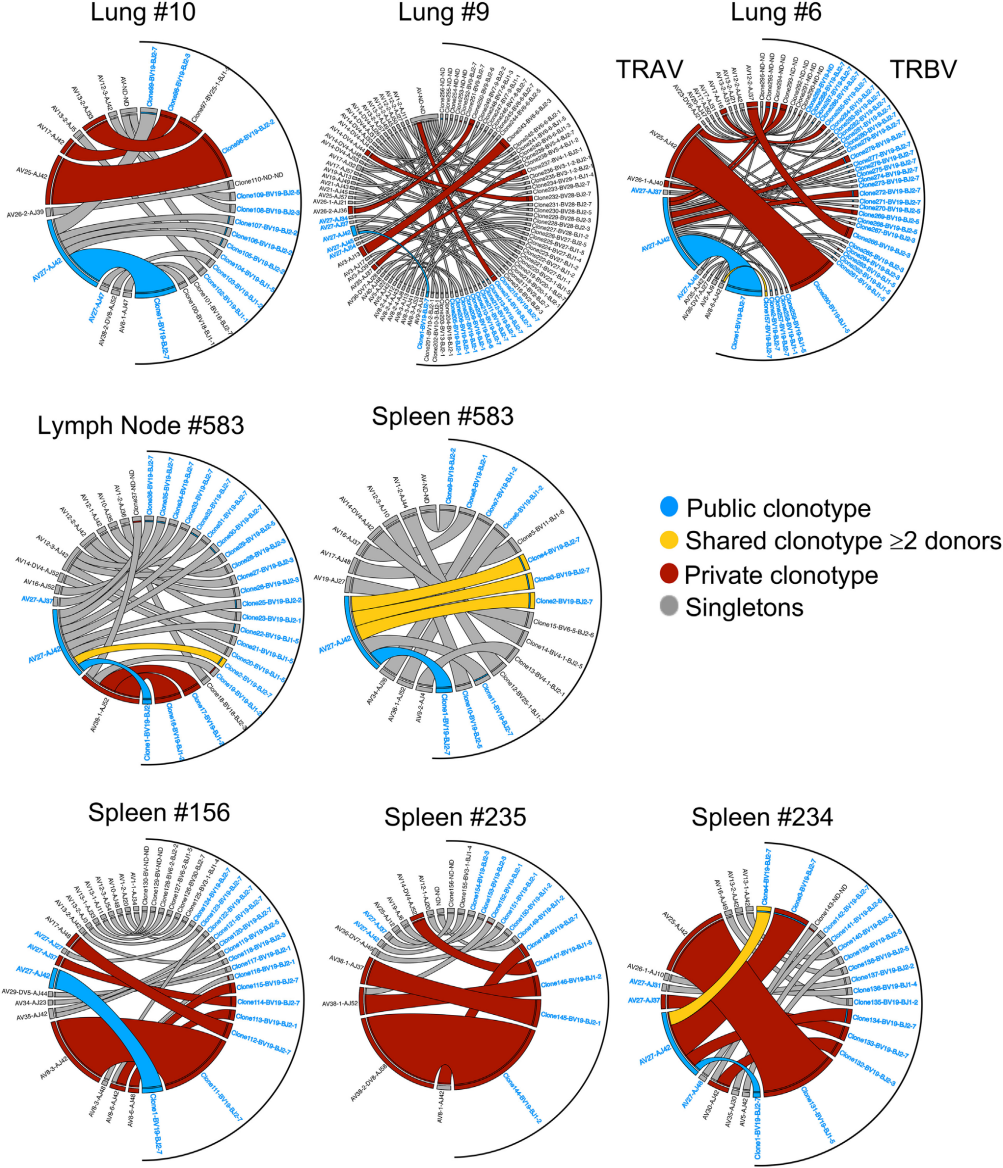
Clonotype frequency of tissue A2^+^M1_58_-specific CD8^+^ T cell receptors (TCRs). Circos plots showing the distribution of TRA-TRB paired clonotypes across tissues (lung, spleen, and lymph node). Each segment defines individual clonotypes and width of segment correlates to frequency of the clonotype. Public clonotype is in blue segments, shared clonotypes between donors are in yellow, private or non-shared clonotypes observed more than once are in maroon, or only once as singletons in gray. For clonotypes having TRBV19/TRAV27 gene usage, the labels have been highlighted in blue. Circos plots were generated using Circos package (51).

To dissect A2^+^M1_58_^+^ TCRαβ signatures within human tissues, we utilized tetramer staining together with single-cell sorting and multiplex RT-PCR approach (7, 36, 40, 41, 44) for spleens (*n* = 3), lungs (*n* = 4), and LN (*n* = 1), with spleens and LN following the TAME protocol and gating strategy, as shown in Figure S1 in Supplementary Material. Our data showed that the composition and distribution of A2^+^M1_58_^+^ TCRαβ signatures within the human tissues (Table 2) was similar to that of TCRαβ clonotypes within peripheral blood of healthy individuals (36, 48). The vast majority of TCRαβ clonotypes in tissues utilized TRBV19 (87 ± 16%) and TRAV27 (36.8 ± 25%) (Table 2; Figures 2 and 3), similar to what has been observed in peripheral blood from healthy adults, with TRBV19 (100%) and TRAV27 (70 ± 21%) (36). The presence of the public TRBV19/TRAV27 TCRαβ was also observed across the tissues, with a mean of 13% (range 2–23%), detected in all the samples tested with the exception of one spleen (#234; Figures 2 and 3; Table 2), compared to a mean of 48% (15–67%) in healthy adults’ blood (36). Our data show, for the first time, that the distribution of human influenza-specific TCRαβ clonotypes in human tissues closely resembles TCRαβ repertoires in peripheral blood of healthy adults.

**Table 2:** Paired TRBV-TRBJ/TRAV-TRAJ clonotype frequencies of A2^+^M1_58_ TCRs in human tissues.

TRBV	TRBJ	CDR3β	TRAV	TRAJ	CDR3α	SP156	SP234	SP235	LN583	SP583	LG10	LG9
^#^BV19	BJ2-7	CASSIRSSYEQYF	AV27	AJ42	CAGGGSQGNLIF	6		10	8		20	
BV19	BJ2-7	CASSIRSSYEQYF	AV8-3	AJ42	CAVGGDGGSQGNLIF	56						
BV19	BJ2-1	CASSIRASGVEQFF	AV8-6	AJ48	CALSGVGGFGNEKLTF	6						
BV19	BJ2-7	CASSIRASYEQYF	AV27	AJ37	CAGASGNTGKLIF	6						
BV19	BJ2-7	CASSIRSGYEQYF	AV8-6	AJ42	CAVPGSQGNLIF	6						
BV19	BJ2-1	CASSIRASGVEQFF	AV8-6	AJ42	CAVGGDGGSQGNLIF	3						
BV19	BJ2-5	CASSTRSGETQYF	AV13-2	AJ42	CAENLGGGSQGNLIF	3						
BV19	BJ2-7	CASSFRSSYEQYF	AV35	AJ42	CAGQLGGGSQGNLIF	3						
BV19	BJ2-7	CASSIRSSYEQYF	AV17	AJ42	CAVGGDGGSQGNLIF	3						
BV19	BJ2-7	CASSIRSSYEQYF	AV35	AJ42	CAGQLGGGSQGNLIF	3						
BV19	BJ2-7	CASSIRSTYEQYF	AV27	AJ27	CAGPATNAGKSTF	3						
BV3-1	BJ1-4	CASSQFNEKLFF	AV13-2	AJ3	CAETPYSSASKIIF	3						
BV6-3	BJ2-2	CASSQVLGLSVTGELFF	AV12-3	AJ54	CATQGAQKLVF	3						
BV19	BJ1-2	CASSQGAYGYTF	AV38-2/DV8	AJ58	CAYRSPKETSGSRLTF		57					
BV19	BJ2-1	CASSMVGGSYNEQFF	AV38-1	AJ37	CAFGHGSSNTGKLIF		14					
BV19	BJ1-2	CASSQGAYGYTF	AV38-1	AJ52	CAFMTNAGGTSYGKLTF		7					
BV19	BJ1-5	CASSIYSNQPQHF	AV14/DV4	AJ52	CAMREDPGGTSYGKLTF		7					
BV19	BJ1-2	CASSQGAYGYTF	AV38-1	AJ52	CAFMKGAGGTSYGKLTF		4					
BV19	BJ2-3	CASSIRSADTQYF	AV27	AJ42	CAGAGGGSQGNLIF		4					
BV19	BJ2-7	CASSIRSAYEQYF	AV8-1	AJ42	CAVGGYGGSQGNILF		4					
BV19	BJ2-7	CASSIRSSYEQYF	AV8-1	AJ42	CAVGGYGGSQGNILF		4					
BV19	BJ1-5	CASSGRSSQPQHF	AV25	AJ42	CAGPGSQGNLIF			35				
BV19	BJ2-7	CASSIRSAYEQYF	AV27	AJ42	CAGGGSQGNLIF			13				
BV19	BJ2-7	CASSVRSSYEQYF	AV27	AJ42	CAGAGSQGNLIF			6		8		
BV19	BJ2-3	CASSTRSTDTQYF	AV30	AJ42	CGTDEAGGSQGNLIF			6				
BV19	BJ2-7	CASSIRSAYEQYF	AV27	AJ37	CAGGGSSNTGKLIF			6				
BV19	BJ1-2	CASSSGSYGYTF	AV27	AJ42	CAGAGSQGNLIF			3				
BV19	BJ2-2	CASSGRAAGELFF	AV27	AJ31	CAPAPDARLMF			3				
BV19	BJ2-5	CASSIRAGETQYF	AV13-2	AJ42	CAENMGGGSQGNLIF			3				
BV19	BJ2-5	CASSKRGAETQYF	AV5	AJ42	CAENAGGGSQGNLIF			3				
BV19	BJ2-5	CASSQRSQETQYF	AV13-1	AJ42	CAASGGGSQGNLIF			3				
BV19	BJ2-6	CASSPFGANVLTF	AV35	AJ30	CAGQNINMNRDDKIIF			3				
BV19	BJ2-7	CASSIRSSYEQYF	AV27	AJ42	CAGAGSQGNLIF			3				
BV19	BJ1-2	CASSIGVYGYTF	AV38-1	AJ52	CAFMKDAGGTSYGKLTF				12			
BV19	BJ1-2	CASSIGSYGYTF	AV38-1	AJ52	CAFMKDAGGTSYGKLTF				8			
BV18	BJ2-3	CASSPLSGRVTDTQYF	AV1-2	AJ36	CAVRDRGKGQTGANNLFF				4			
BV19	BJ1-2	CASSIGAHGYTF	AV38-1	AJ52	CAFMKGPGGTSYGKLTF				4			
BV19	BJ1-5	CASSIRSSQPQHF	AV12-3	AJ42	CAIDLGGGSQGNLIF				4			
BV19	BJ1-5	CASSLYSNQPQHF	AV27	AJ42	CAGGGFADYGGSQGNLIF				4			
BV19	BJ2-1	CASSIRSSNEQFF	AV12-1	AJ42	CVVNYGGGSQGNLIF				4			
BV19	BJ2-1	CASSKFSINEQFF	AV14/DV4	AJ31	CAMREDNGARLMF				4			
BV19	BJ2-2	CASSIRSSGELFF	AV27	AJ42	CAGASGGSQGNLIF				4			
BV19	BJ2-3	CASSFGSTDTQYF	AV12-1	AJ42	CAGDGGGGSQGNLIF				4			
BV19	BJ2-3	CASSFRSTDTQYF	AV12-1	AJ42	CAGDGGGGSQGNLIF				4			
BV19	BJ2-5	CASSIRAGETQYF	AV12-3	AJ42	CAMSGDGGSQGNLIF				4			
BV19	BJ2-7	CASSIRAAYEQYF	AV27	AJ42	CAVALGGGSQGNLIF				4			
BV19	BJ2-7	CASSIRASYEQYF	AV12-3	AJ42	CAMSGDGGSQGNLIF				4			
BV19	BJ2-7	CASSIRSAYEQYF	AV27	AJ42	CAGVDGGSQGNLIF				4			
BV19	BJ2-7	CASSIRSSYEQYF	AV16	AJ52	CALNAGGTSYGKLTF				4			
BV19	BJ2-7	CASSIRSSYEQYF	AV27	AJ37	CAGSFGSSNTGKLIF				4			
BV19	BJ2-7	CASSMRSSYEQYF	AV27	AJ42	CAGAGSQGNLIF				4			
BV19	BJ2-7	CASSTRSSYEQYF	AV27	AJ42	CAGAGSQGNLIF				4			
BV19	BJ2-7	CASSVRSSYEQYF	AV27	AJ42	CAGGGSQGNLIF				4			
BV19	BJ2-7	CASSIRSAYEQYF	AV27	AJ42	CAGAGSQGNLIF					17		
BV19	BJ1-2	CASSIGVYGYTF	AV38-1	AJ52	CAFMKGAGGTSYGKLTF					8		
BV19	BJ1-2	CASSTGLYGYTF	AV34	AJ26	CGAAHNYGQNFVF					8		
BV19	BJ2-1	CASSIRSGYEQFF	AV16	AJ37	CALKRSNTGKLIF					8		
BV19	BJ2-5	CASSIRAGETQYF	AV12-3	AJ10	CASSGVGGRQNNLIF					8		
BV19	BJ2-7	CASSIRSAYEQYF	AV27	AJ42	CGGGGSQGNLIF					8		
BV19	BJ2-7	CASSIRSSYEQYF	AV27	AJ42	CAGASPGGYGGSQGNLIF					8		
BV25-1	BJ1-2	CASYTSDYGYTF	AV1-2	AJ44	CAVRDRVDGTASKLTF					8		
BV4-1	BJ2-1	CASSPSLAGYGNEQFF	AV9-2	AJ4	CALSDSGYNKLIF					8		
BV9	BJ1-6	CAMYLCASSLFGSPLHF	AV17	AJ48	CATHNFGNEKLTF					8		
BV19	BJ2-2	CASSARSTGELFF	AV25	AJ42	CAGNYGGSQGNLIF						24	
BV25-1	BJ1-5	CASSAQANQPQHF	AV13-2	AJ33	CAEGARDSNYQLIW						12	
BV19	BJ2-3	CASSFRSTDTQYF	AV17	AJ42	CATDGGGGSQGNLIF						8	
BV19	BJ2-7	CASSSRSSGEQYF	AV27	AJ42	CAGAYGGSQGNLIF						8	
BV18	BJ1-1	CASSQTGLNTEAFF	AV27	AJ47	CAMSPMEYGNKLVF						4	
BV18	BJ2-7	CASSSVGSPYEQYF	AV8-1	AJ47	CAAWASSEGNKLVF						4	
BV19	BJ1-1	CASRINPGGADEAFF	AV13-2	AJ5	CAENAPLGRRALTF						4	
BV19	BJ2-2	CASSARSTGELFF	AV27	AJ42	CAGGGSQGNLIF						4	
BV19	BJ2-2	CASSGRATGELFF	AV27	AJ42	CAGEGGSQGNLIF						4	
BV19	BJ2-2	CASSTRSTGELFF	AV27	AJ42	CAGAGGGSQGNLIF						4	
BV19	BJ2-5	CASSTRATETQYF	AV12-3	AJ42	CAMTGDGGSQGNLIF						4	
BV6-6	BJ2-3	CASSYLAGEITDTQYF	AV3	AJ13	CAVRDISVSGGYQKVTF							11
BV28	BJ2-7	CASSSPKGAKYEQYF	AV14/DV4	AJ44	CAMREVPRGASKLTF							6
BV4-1	BJ2-1	CAVLVDPYNEQFF	AV35	AJ37	CAGPSNTGKLIF							6
BV9	BJ2-7	CASSVDPAGGSSYEQYF	AV26-1	AJ36	CILKTGANNLFF							6
BV10-3	BJ2-1	CAISESTAHSYNEQFF	AV3	AJ17	CAVRDPFKAAGNKLTF							3
BV13	BJ2-1	CATDARVGNTGELFF	AV17	AJ57	CATDAWGGSEKLVF							3
BV18	BJ2-1	CASSPEAGVSNTEAFF	AV8-2	AJ12	CAVSAVMDSSYKLIF							3
BV19	BJ2-6	CASSIVIVAGANVLTF	AV12-1	AJ27	CAVNTNAGKSTF							3
BV19	BJ2-7	CASGQGAGEQYF	AV21	AJ43	CAVREYNNNDMRF							3
BV19	BJ2-7	CASSIRSSYEQYF	AV27	AJ42	CAAGGSQGNLIF							3
BV19	BJ2-7	CASSTRSSYEQYF	AV27	AJ42	CAGGGSQGNLIF							3
BV19	BJ2-7	CASSVRSSYEQYF	AV27	AJ37	CAGAHGSSNTGKLIF							3
BV2	BJ2-3	CASSGSDTQYF	AV13-2	AJ57	CAENMFQGGSEKLVF							3
BV20-1	BJ2-1	CSARDTSGSYNEQFF	AV27	AJ34	CAGRLWTDKLIF							3
BV27	BJ1-2	CASRLHPGHMSYTF	AV14/DV4	AJ26	CAMNDGQNFVF							3
BV27	BJ1-5	CASSLKTHYSNQPQHF	AV19	AJ13	CAGDSGGYQKVTF							3
BV27	BJ2-5	CASSISGGPGETQYF	AV5	AJ42	CAGGGSQGNLIF							3
BV28	BJ2-3	CASIRGLAGVRTDTQYF	AV3	AJ39	CAVRAFYAGNMLTF							3
BV28	BJ2-3	CASSALVLYATDTQYF	AV21	AJ45	CAVVRGGADGLTF							3
BV28	BJ2-5	CASSLGAGFLQETQYF	AV14/DV4	AJ48	CAMREGQTNFGNEKLTF							3
BV28	BJ2-7	CASSLPKTINSEQYF	AV8-2	AJ8	CAIGFQKLVF							3
BV3-1	BJ2-1	CASSLQISIAGVSYNEQFF	AV12-1	AJ50	CAVKTSYDKVIF							3
BV5-4	BJ2-7	CASSPFQGSYEQYF	AV8-3	AJ53	CAVSERESGGSNYKLTF							3
BV6-6	BJ1-5	CASSYSRPGLSNQPQHF	AV27	AJ45	CAGGRHSGGGADGLTF							3
BV6-6	BJ2-5	CASSYFGLAFQETQYF	AV27	AJ54	CAGGGIQGAQKLVF							3
BV6-6	BJ2-7	CASSGLAGARNEQYF	AV8-2	AJ43	CVVSEGVTEDMRF							3
BV7-8	BJ2-7	CASTPHRGPSYEQYF	AV19	AJ49	CALSEANTGNQFYF							3
BV7-9	BJ1-3	CASTPSSGSAGNTIYF	AV14/DV4	AJ4	CAMRRPSGGYNKLIF							3
BV7-9	BJ2-2	CASSSPPDRGPNTGELFF	AV8-3	AJ13	CAVGYSGGYQKVTF							3

CDR3αβ pairs						36	28	31	25	12	25	35

*^#^Highlighted in blue is the public clonotype observed in adult peripheral blood A2^+^/M1_58_ TCRs*.

*TCRs, T cell receptors; CDR3, complementarity-determining region 3*.

**Figure 4:**
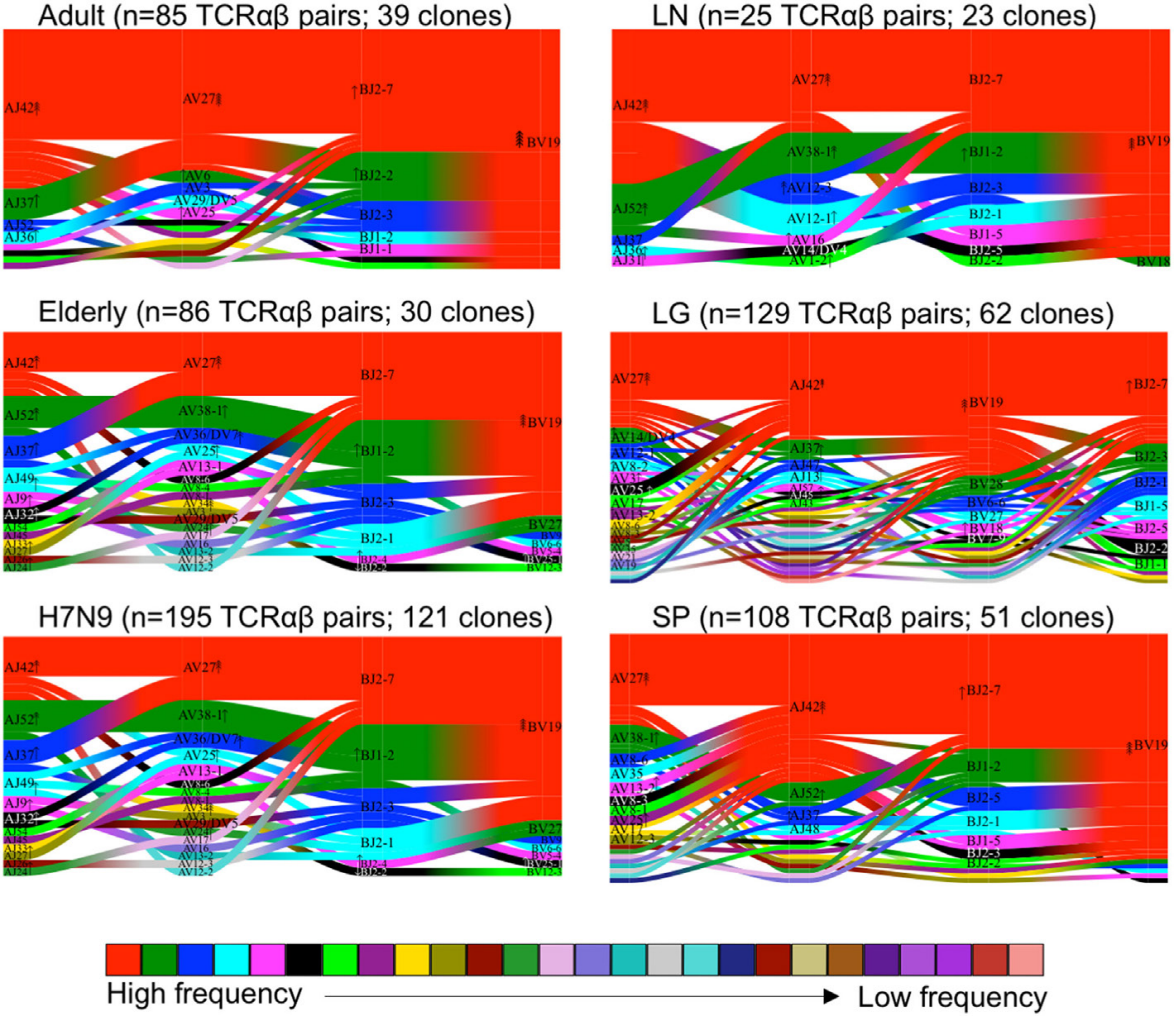
Maintenance of clonal diversity across age group and tissue compartment. Gene segment usage and pairing landscapes are shown for each group. Each clonotype is assigned the same vertical length irrespective of clonotype size. Each vertical stack reflects the V and J gene segment usage and pairing is shown by curved connecting lines. Genes are ranked in color by the frequency distribution with red being the highest frequency, followed by green, dark blue, aqua, magenta, black, and thereafter. Enrichment or depletion of gene usage is indicated by up or down arrows, respectively, where one arrowhead correlates to a two-fold increase or decrease.

We further performed comprehensive analyses of A2^+^M1_58_^+^CD8^+^ TCRαβ repertoire diversity across human tissues using our newly generated dataset in comparison to our previously published datasets from peripheral blood TCRαβ clonotypes found in adults (*n* = 3) (36), elderly (*n* = 3) (40), H7N9-infected patients (*n* = 3) (7), and one lung tissue (LG6) (41). Our total dataset comprised of 628 paired TCRαβ sequences that was parsed through TCRdist algorithm (38). TCRdist was used to calculate a normalized Jensens–Shanon divergence to measure the gene segment usage by comparing the probability occurrence of our A2^+^M1_58_^+^ epitope-specific TCRs against a background dataset of other epitope-specific TCRs (38). Gene segment pairing was represented as average-linkage dendrograms (Figure 4), where each clonotype presented a constant vertical height, and the gene segment within each clonotype was joined by curve segments to neighboring genes. Thickness of the segment indicated the number of unique clones sharing the same gene, with the TRBV segment almost completely comprising of TRBV19 (in red) across all peripheral blood adult/elderly donors and spleen and LN samples. With respect to background TCRs (38), the analysis of A2^+^M1_58_^+^ TCR repertoire indicated 16-fold enrichments for TRBV19, 8- to 16-fold enrichments for TRAV27 gene usage, 2-fold enrichments for TRBJ2-7, and 4- to 8-fold enrichments for TRAJ42, with each arrow corresponding to a 2-fold increase or decrease (Figure 4). Overall, we found no striking differences for the most preferred A2^+^M1_58_^+^CD8^+^ gene usage represented by TRBV19/TRBJ2-7/TRAV27/TRAJ42 (36). However, A2^+^M1_58_^+^CD8^+^ TCRα-chain within healthy elderly, elderly H7N9-infected patients, spleen, and lung donors displayed increased variability within Vα and Jα gene usage. Thus, our data show that the TCRαβ diversity within A2^+^M1_58_^+^CD8^+^ T cells results from a diverse gene usage at Vα, Jα, and Jβ locus.

### Conserved CDR3α and CDR3β Length Usage in Human Tissues

**Figure 5:**
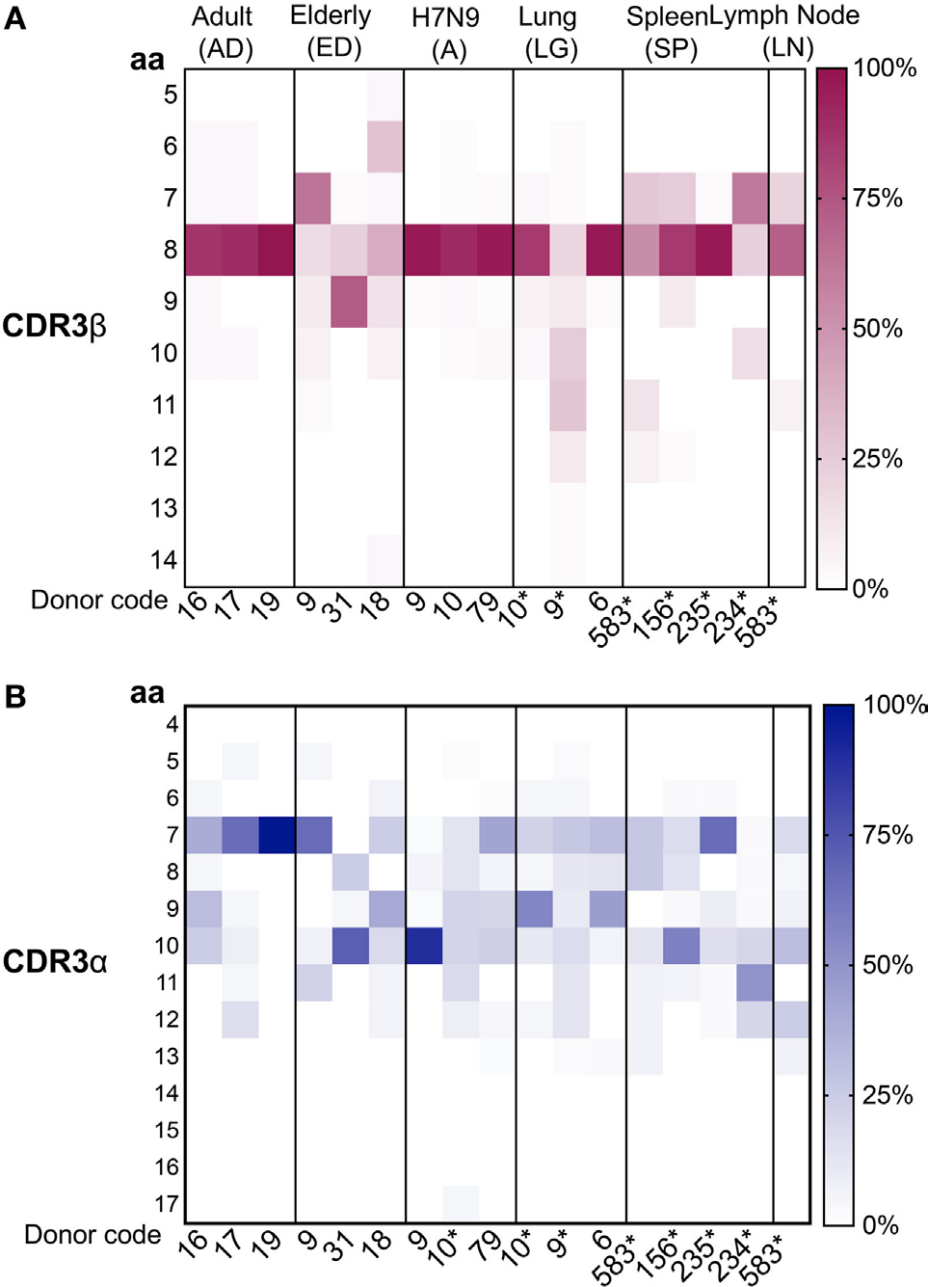
Conserved complementarity-determining region 3 (CDR3)β and diverse CDR3α amino acid (aa) length usage. Distribution of **(A)** CDR3β and **(B)** CDRα aa length usage across adult, elderly, lung, spleen, lymph node (LN) (*n* = 1), and H7N9 hospitalized patients (all groups have *n* = 3 unless specified). CDR3 lengths for donors marked by “*” were generated from this study. Data from unmarked donors were taken from our previous studies as described [adult (36), elderly (40), H7N9 hospitalized patients (7), and lung (41)]. Heatmaps were generated using Matplotlib package (50).

As CDR3 loops play an important role in p-MHC-I contact (58), we next examined the CDR3 aa length within A2^+^M1_58_^+^CD8^+^ TCRs in human tissues, taking into consideration the size of each clonotype to highlight the contribution of the expanded clonotypes. As shown by our previous data, A2^+^M1_58_^+^CD8^+^ TCRβs within the peripheral blood of adults (36) and H7N9-infected patients (7) utilized mainly CDR3β lengths of eight aa, representing 92 ± 7 and 94 ± 3.5%, respectively. Such strong bias toward CDR3β lengths of eight aa was also found in human tissues (mean 66.3 ± 31%) (Figure 5A) but was greatly reduced in healthy elderly donors (40). CDR3α loops on the other hand encompassed a wide range of CDR3α lengths, displaying specific variations across different datasets (Figure 5B). Cumulative analysis across different groups showed that only ED#9 (66%) and SP#235 (67%) showed the preferred seven aa length as reported for adult (68 ± 30.5%) A2^+^M1_58_^+^CD8^+^ TCRα chains, while the remaining donors from healthy elderly individuals, H7N9-infected elderly patients and tissues had longer CDR3α lengths (Figure 5B). Similar observations were reported by Gil et al. for A2^+^M1_58_^+^CD8^+^ TCRα chains within elderly donors (34). Interestingly, CDR3α chains with seven aa in the elderly maintained a biased pairing with TRAV27 and TRAJ42 genes. Thus, our paired TCRαβ analyses highlight the plasticity of TRBV19 genes with a preferential CDR3β length of eight aa to pair with diverse TRAV regions with CDR3α’s across different lengths.

### Large Dominant Private TCRαβ Clusters Dominate A2^+^M1_58_^+^CD8^+^ T Cells in the Elderly Donors

**Figure 6:**
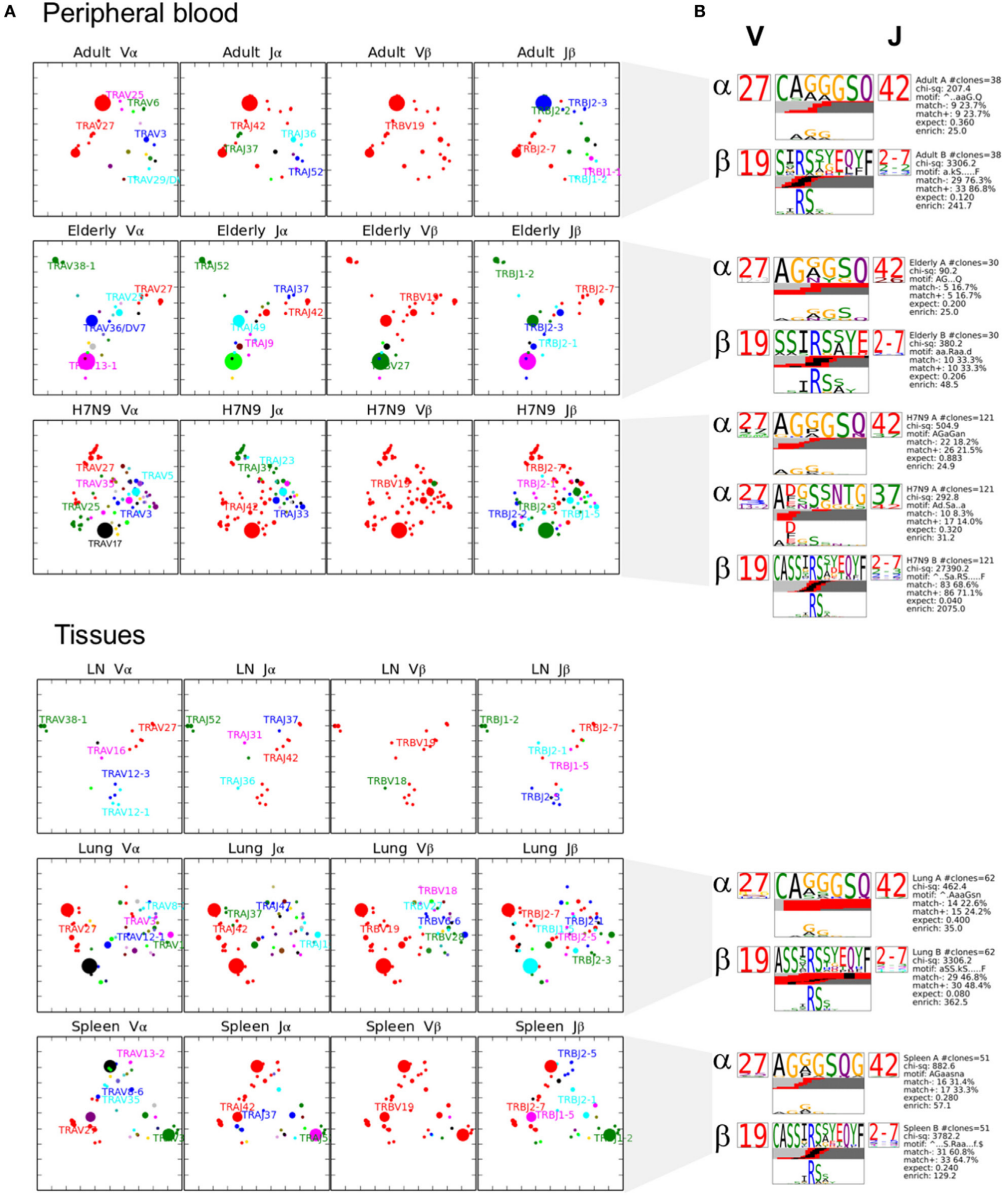
Private clonotypes dominate elderly T cell receptors (TCRs), while public TCRs are represented in blood and across different tissues. **(A)** 2D kernel principal component analysis (kPCA) projections for healthy adult, healthy elderly, elderly H7N9 hospitalized patients, lung, lymph node (LN) (*n* = 1), and spleen (all groups have *n* = 3 unless specified). Size of the clone correlates with clonotype size and color correlates to gene usage. Most prevalent gene usages are mentioned within the plots matching with clonotype color. Each row represents group and each column is the same 2D kPCA projection of the four gene segment usage (Vα, Jα, Vβ, and Jβ). **(B)** Using TCRlogo in TCRdist algorithm, top-scoring complementarity-determining region 3 (CDR3)β and CDR3α motifs are shown for each group, except for LN. V and J gene usage are indicated left and right of motifs, respectively, and bottom panel highlights the motif enriched by calculating against a background dataset of naïve non-A2^+^M1_58_-specific CD8^+^ TCRs.

**Figure 7:**
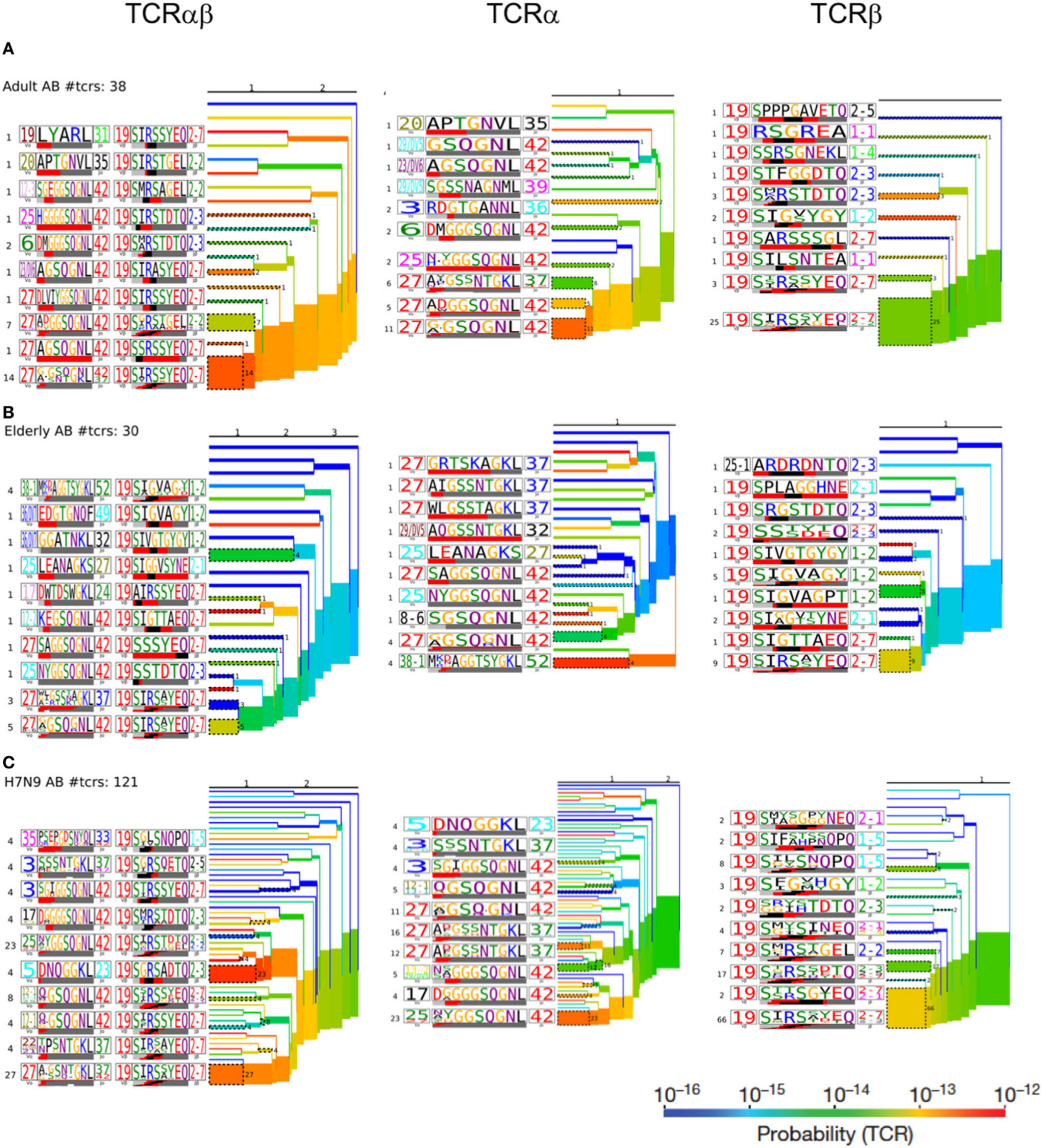

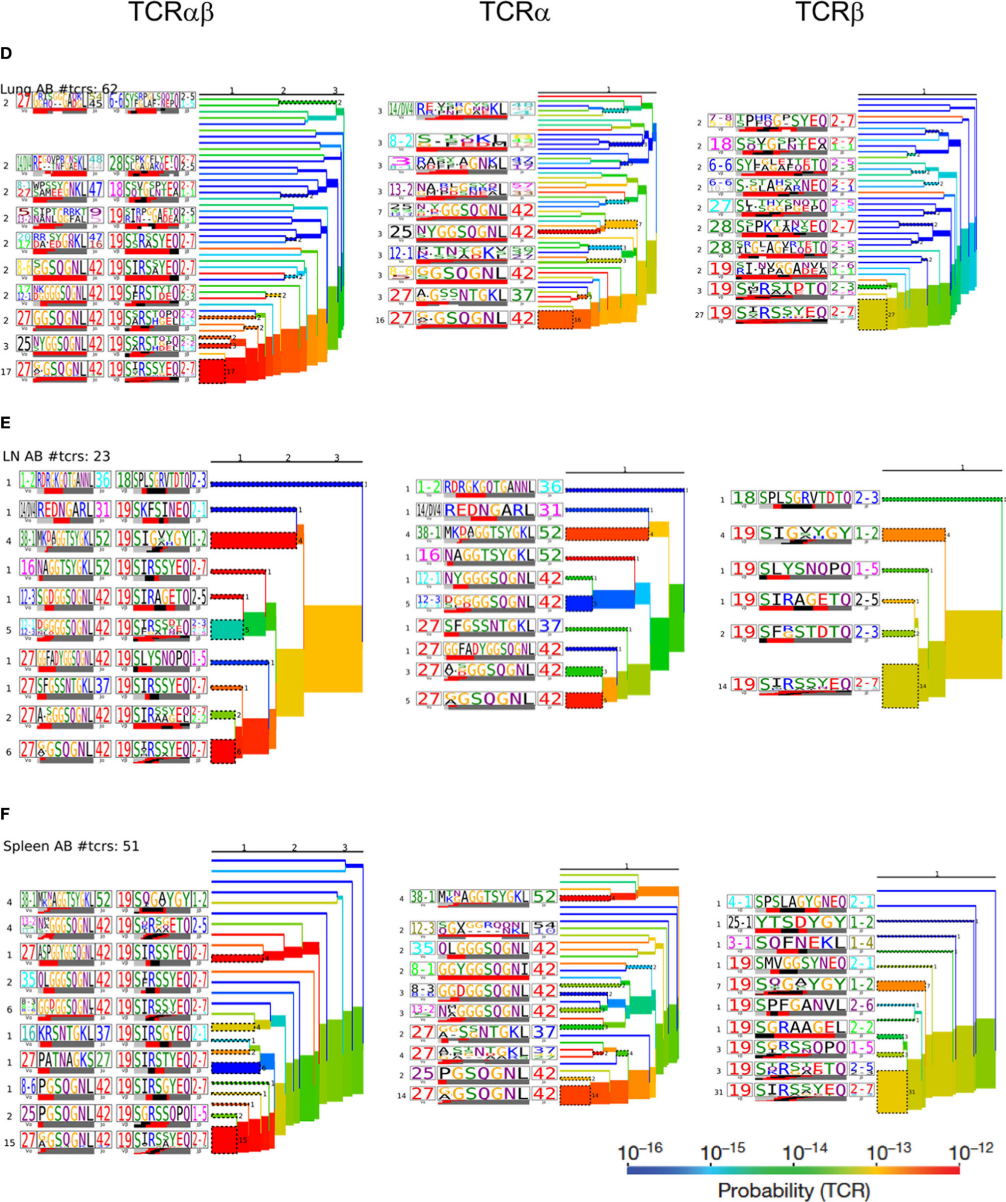
Hierarchal clustering of T cell receptors (TCRs) highlights diversity of aging A2^+^M1_58_-specific CD8^+^ TCRs: TCRαβ, TCRα, and TCRβ clustering along with corresponding TCRlogos for **(A)** adult, **(B)** elderly adult, **(C)** elderly H7N9 patients, **(D)** lungs, **(E)** lymph nodes, and **(F)** spleen. Number on the branches and next to TCRlogos depicts number of TCRs contributing to the cluster. Color of the branches indicates the TCR probability generation scores.

To further understand the distribution of A2^+^M1_58_^+^CD8^+^ TCRs based on the level of similarity within the CDR3 regions, TCRdist (38) computes the similarity-weighted hamming distance between given TCRs in a group and presents the two-dimensional kernel principal component analysis (a non-linear form of PCA) of TCR clusters reflecting the clonal expansions and gene usage. TCRdist also allows identification of top-scoring CDR3α and CDR3β motifs crucial in recognizing the p-MHC-I complex. TCRdist quantifies all these by comparing a given set of epitope-specific TCRs against a background set of publicly available non-epitope-specific TCR dataset. Using TCRdist, we observed that adult, H7N9-infected elderly and tissue samples exhibited TCR Vβ clusters dictated by large clonotypes with TRBV19 usage (Figure 6A, in red), while, the dispersion in TCR Vα clusters resulted in the presence of large clonotypes of non-TRAV27 usage (Figure 6A). Conversely, elderly TCR clusters highlighted the presence of large private clonotypes with decreased TRBV19/TRAV27 gene usage (Figure 6A, second row panels). However, despite such reduced frequency of TRBV19 and TRAV27 gene usage in elderly donors, analysis of the key “RS” motif in TRBV-CDR3β and the glycine-rich “GG” motif in TRAV27-CDR3α clearly demonstrates a preservation of those conserved CDR3 residues across age groups, tissues and following influenza virus infection (Figure 6B). The patterns observed in our TCR clusters were further complemented by our TCRlogo summary of CDR3 aa sequences and corresponding gene frequencies (Figure 7). As compared to the adult and H7N9-infected elderly (Figures 7A,C; higher proportion of red shades), elderly adult TCRs had lower probability scores (probability of the TCR being generated from germ-line TCRs; blue shades) (Figure 7B). Similarly, tissues also presented a large cluster with high probability scores (Figures 7D–F). Observations at a single chain level further showed that elderly A2^+^M1_58_^+^CD8^+^ TCRs were composed of more diverse and rarer TCRs, as shown by high proportion of TCRs with low probability scores (Figure 7). However, elderly donors TCRs still presented “RS” motif containing TRBV19 (five distinct TCRs) pairing with “GG” containing diverse TRAV27 (five distinct TCRs). The representation of “RS” and “GG” motif was more pronounced in adult, H7N9 patients, and tissues (Figure 7). Therefore, our analyses highlight that despite large clonal expansions of private TCRαβ clonotypes found in the elderly individuals (36, 37), the key motifs are still present in the memory A2^+^M1_58_^+^CD8^+^ T cell pool, but with much lower probability for the elderly individuals. Most striking is that these key motifs are still efficiently recruited following severe influenza virus infection in the elderly patients (7).

### A2^+^M1_58_-Specific Public CDR3βs Form Highly Connected Similarity Networks

**Figure 8:**
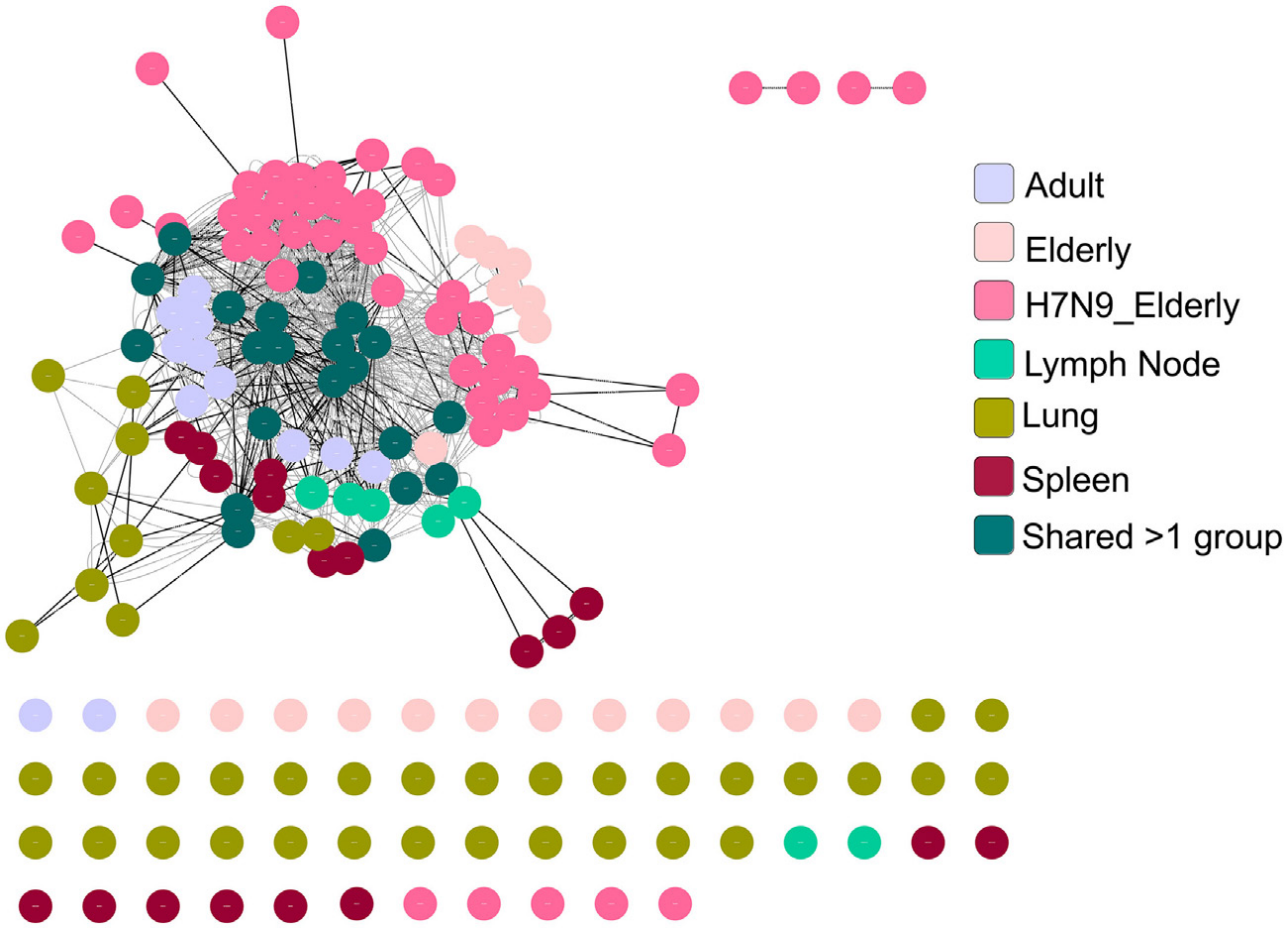
A2^+^M1_58_-specific complementarity-determining region 3 (CDR3)βs forms a dense cluster comprising public T cell receptors (TCRs). TCR network organization of CDR3β from A2^+^M1_58_-specific CD8^+^ TCR repertoire from adult, elderly, elderly H7N9 patients, spleen, lymph node, and lung to study the level of sequence similarity within and between donors. Black lines are global interactions (a pair of TCRs that share the same length CDR3 and differ by less than a certain number of amino acid) and the gray lines are local interactions. Node (dot) represent unique clonotype, edge is the global (black line) or local (gray line) interactions between the nodes. Colors of the node indicate the group to which the node belongs as shown in the legend.

As the public (or near-public) TCRβ clonotypes are a key signature of the A2^+^M1_58_^+^CD8^+^ TCRαβ repertoire with heavy bias for TRBV19, we further dissected in-depth CDR3β clusters across age groups, tissues, and following influenza virus infection. Using GLIPH, we focused on TCRβ chains by identifying enriched motifs independent of the V-J chain usage (39). We generated a network of all CDR3β regions defined as nodes connected by neighboring (by sequence similarity) CDR3β nodes, which have global or local interactions or no interactions at all (defined as singletons). We then visualized the A2^+^M1_58_^+^ CDR3β network using the clusterMaker plugin of Cytoscape (59) across age groups, anatomical locations, and during acute influenza virus infection. Our network organization of CDR3β regions (628 sequences) across all the datasets demonstrated that the most abundant public CDR3β sequences comprising the “RS” motif formed a dense network with many interconnecting nodes within and between the different donor groups (Figure 8). For example, nodes representing shared TCRβ clonotypes between groups were “most-dense” and found mainly in the center of the cluster. Rarer private CDR3β sequences sharing no motifs formed singletons (disconnected nodes), while CDR3β clonotypes with low level of motif sharing clustered around the edges of the public network, highly evident for the large private clonotypes observed in our healthy elderly cohort.

**Figure 9:**
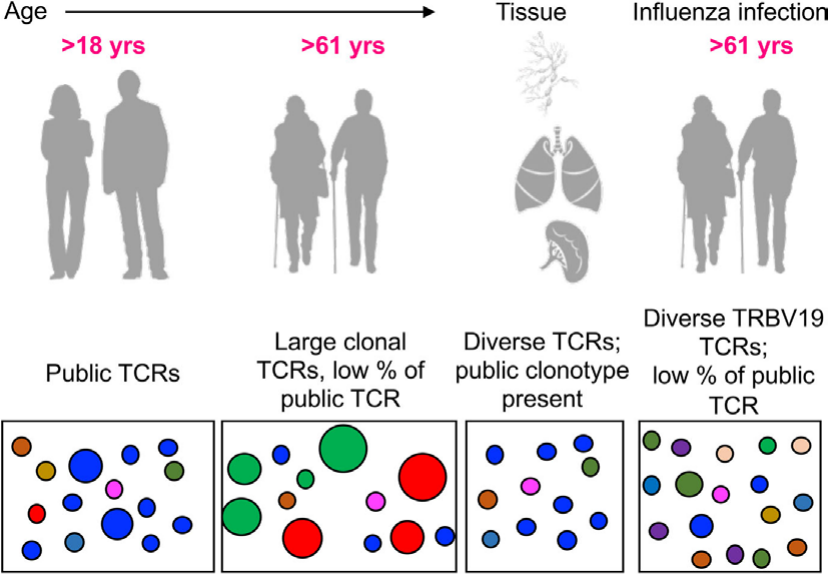
Modeling the dynamic changes of A2^+^M1_58_-specific CD8^+^ T cell receptor (TCR) repertoires with age, tissue location, and during influenza infection. A conceptual figure summarizing evolution of A2^+^M1_58_^+^ CD8^+^ TCR repertoires in young adults (with large public clonotypes depicted in blue), elderly adults (displaying loss of public clonotypes and presence of large private clonotypes in green and red), tissues (with public clonotypes along with diverse set of TCRs), and influenza-infected elderly adults (displaying diverse TRBV19s and public clonotypes).

Taken together, our analysis showed that aging had the greatest impact on the A2^+^M1_58_^+^ memory CD8^+^ T cell repertoire, with a considerable loss of public TCRαβ signatures being paralleled with large clonal expansions of private TCRαβ clonotypes. TCRαβ analysis during acute H7N9 infection in elderly donors revealed that the immune system was capable of recruiting diverse TRBV19, rather than largely expanded private TCRs, in spite of considerably low frequency of public TCRs. Memory antigen-specific TCRs in tissues displayed the main features of the public TCRαβ clonotypes along with the presence of other diverse TCR repertoires, as summarized in Figure 9. Overall, this study establishes the framework for understanding antigen-specific TCRs across the human life span (young and old) and different anatomical locations, thus providing a clearer picture on the antigen-specific “TCRome,” with implications for the rational design of T cell-mediated vaccines and immunotherapies.

## Discussion

Preexisting memory CD8^+^ T cells can recognize internal conserved peptides derived from influenza viruses, irrespectively of the strain, thus mount effective immune responses toward newly emerging viruses (8–10, 60). This can be exemplified by a positive correlation between the presence of functional influenza-specific CD8^+^ T cells and rapid recovery in patients hospitalized with severe avian H7N9 virus (7, 10). Several factors can affect virus-specific CD8^+^ T cell responses, including numbers and quality of influenza-specific T cells, aging, disease, as well as the composition and diversity of TCRαβ repertoire against a given epitope, which has been well documented in mouse studies (54, 61, 62). With respect to influenza infection, human studies have mostly focused on antigen-specific CD8^+^ T cells residing in peripheral blood (11, 34, 42, 48). Our study presents the first data on influenza-specific A2^+^M1_58_^+^CD8^+^ T cell repertoires across different human tissue compartments, including LNs, spleens, and lungs, and provides comprehensive analyses on temporal dynamics and maintenance of epitope-specific CD8^+^ T cells with aging, influenza virus infection, and across different tissue compartments.

We dissected A2^+^M1_58_^+^CD8^+^ T cells across young and elderly age groups, during severe influenza infection and within human lung tissues from our published studies (7, 36, 40, 41), along with new TCRαβ data generated from additional tissues sampled across different anatomical sites. Using human A2^+^M1_58_-tetramer magnetic enrichment or direct-staining (for lungs) for isolation of epitope-specific CD8^+^ T cells directly *ex vivo*, we observed the presence of A2^+^M1_58_^+^CD8^+^ T cells in human spleen, mesenteric/pancreatic LNs, and in the lung. The frequency of these cells was comparable to memory or effector A2^+^M1_58_^+^CD8^+^ T cells found in peripheral blood. The presence of A2^+^M1_58_^+^CD8^+^ T cells in the human lung using tetramer staining was first reported very recently by our group, and for one donor (LG6), TCR analysis was previously performed showing a high degree of TCRαβ clonotype sharing between different tissue-resident T cell subsets (41). Here, we have performed further TCRαβ analysis on two additional A2^+^ lung donors as well as source different human tissues from deceased adults for our study. Hence, to the best of our knowledge, our study is the first to isolate and characterize influenza-specific CD8^+^ T cells from multiple different human tissues. Most strikingly, the presence of A2^+^M1_58_^+^CD8^+^ T cells in LNs and secondary tissues supports the idea that human spleen and distal LNs serve as reservoirs of antigen-specific memory CD8^+^ T cells. This has been well established in mouse models of local and systemic virus challenge, showing that effector or memory CD8^+^ T cells can traffic to distal tissue during a secondary challenge, irrespectively of location of primary infection, and contribute to the overall immune response on secondary infection (54, 61, 63). Future in-depth studies should highlight the functional relevance of these antigen-specific CD8^+^ T cells in comparison to tissue-resident T cells residing at the site of primary infection (lungs).

Utilizing paired single-cell TCRαβ sequencing technology, we dissected TCRαβ repertoires of A2^+^M1_58_^+^CD8^+^ T cells isolated from tissues and compared those to our published datasets of A2^+^M1_58_-specific TCRαβ repertoires. We performed comparative analyses utilizing TCRdist allowing dissection of gene usage within Vα, Jα, Vβ, and Jβ segments. The A2^+^M1_58_-specific TCRαβ repertoire is characterized by the dominant presence of the TRAV27/TRBV19 TCR signature presenting the public CDR3αβ motif “GGSQGNL”/“SIRSYEQ” (36). In support of our initial observations that the elderly adults had less of the public A2^+^M1_58_ TCR signatures (40), our new analyses encompassing age, tissue compartment, and infection, showed that aging had the greatest impact on memory A2^+^M1_58_^+^ CD8^+^ T cells repertoire, leading to a reduced frequency and lower probability of TCR clonotypes presenting the TRAV27/TRBV19 TCRαβ signatures. Moreover, the presence of TCRs with low generation probability corresponded to the high frequency of private clonotypes and a loss of public clonotypes, thus highly reflected aging-associated changes in TCR repertoires in elderly at a steady state. Importantly, these TRAV27/TRBV19 TCRs display the key motifs for A2^+^M1_58_ epitope recognition, similar to those found in adults, tissues, and influenza-infected patients. Thus, any T cell vaccine development needs to consider strategies to maintain high frequency of the optimal TCRαβ signatures for efficient immunity against influenza infection in the elderly individuals, who are most vulnerable to severe influenza disease.

A striking observation was the variable aa lengths of the CDR3α loop across all the datasets, although longer CDR3α lengths were preferentially observed in the elderly donors, similar to our previous findings (34). Conversely, CDR3β length was consistently maintained at eight aa, with the exception of elderly memory A2^+^M1_58_^+^TCRαβ repertoires (nine aa length preferred). The CDR3α length has been previously shown to affect the p-MHC-I affinity (37). A comparison between the JM22 TCR (public TRAV27/TRBV19 motif “AGSQGNL”/“SIRSYEQ”) with that of F6 TCR (TRAV27/TRBV19 motif “AIGSSNTGKL”/“SIRSYEQ”), both displaying identical CDR3β, showed that longer CDR3α loop reduced the affinity of F6, due to possible high entropic cost in stabilizing the CDR3α loop.

Using GLIPH, our TCRβ network analyses showed that public clonotype and “public-like” clonotypes formed dense clusters, while more private clonotypes formed either independent smaller clusters or remained disconnected. Our observation of a dense network within memory, young versus old, and across different tissue locations suggests the likelihood of structural constraints of diverse TCRs to accommodate recognition of the featureless A2^+^M1_58_ topology. However, antigen recognition is mediated by interactions of both TCRα and TCRβ with the p-MHC-I complex, thus differences observed in the CDR3α length usage could hamper p-MHC-I recognition irrespective of the presence of the “best fit” TCRβ. Future studies are needed to correlate our findings with structural studies and functional characteristics of TCRs displaying restricted TCRβ chain pairing with diverse CDR3α chains.

In our study, we used a single-cell approach for TCRαβ analysis across different age groups and tissue compartments. This approach allowed us to gather data on the paired TCRα and TCRβ signatures obtained from single cells as well as dissect TCRαβ clonotypes in human samples with limited cell numbers. On the other hand, bulk sorted-A2^+^M1_58_^+^CD8^+^ T cells would provide more depth (a higher number of TCR sequences) to the study, however, a major disadvantage of using a bulk TCR approach is its inability to obtain paired TCRαβ chains. Thus, single-cell sequencing techniques identifying paired TCRαβ sequences are a very powerful tool for understanding the contribution of both alpha and beta TCR chains to the overall TCR repertoire in health and disease.

Overall, our study provides comprehensive analyses of A2^+^M1_58_-specific TCRαβ repertoires to understand temporal dynamics of TCRαβ repertoire selection and maintenance across young and aged donors, during human acute influenza infection, at primary site of infection (lungs), and at tissues distal to primary site of infection. Our analyses emphasize the remarkable clonal stability of the A2^+^M1_58_-specific TCRαβ repertoire across different tissue locations and show the greatest impact of aging on influenza-specific TCRαβ repertoire. Future studies are needed to fully understand how aging impacts recruitment of influenza-specific TCRs in the elderly during acute influenza disease. Compilation of similar analyses, for other immunodominant CD8^+^ T cell epitopes across different HLA alleles, across human lifespan and tissues could greatly benefit any T cell-based vaccines and immunotherapies.

## Ethics Statement

This study was approved by the University of Melbourne Human Ethics Committee (ID 1443389.3 and 0931311.5). Human experimental work was conducted according to the Declaration of Helsinki Principles and according to the Australian National Health and Medical Research Council Code of Practice. All donors provided informed written consent for blood donation. Tissues from deceased organ donors were obtained following written informed consent from the next of kin. PMBCs were isolated from heparinized peripheral blood collected from healthy adult volunteers (AD, 22–59 years) at the University of Melbourne and elderly donors (ED, 60 years) recruited at Deepdene Medical Clinic (Deepdene, Australia), or from buffy packs of healthy adult donors (AD, 22–59 years) obtained from Australian Red Cross Blood Service (ARCBS). Peripheral blood from HLA-A*02:01^+^ H7N9-infected patients (A9, A10, and A79) were obtained from Shanghai Public Health Clinic Centre (SHAPHC) under the supervision of SHAPHC Ethics Committee (7). PBMCs were also obtained from one patient hospitalized at the Royal Melbourne Hospital in 2017 with seasonal-influenza virus infection and approved by the Monash Health Human Research Ethics Committee (HREC/15/MonH/64).

## Author Contributions

SS, TN, and KK designed the research. SS, TN, ZW, AP, LW, MK, and LG performed and/or analyzed the research. GW provided lung donor tissue. TL and SM provided spleen and lymph node donor tissues. JX and MR provided influenza patient samples. JC provided elderly adult donor samples. SS, LG, ZW, LL, AP, GW, MK, TN, LW, JC, JR, SG, PT, and KK contributed to manuscript preparation. SS, TN, and KK wrote the manuscript.

## Conflict of Interest Statement

The authors declare that the research was conducted in the absence of any commercial or financial relationships that could be construed as a potential conflict of interest. The handling Editor declared a past co-authorship with one of the authors KK.

## Footnotes

^1^http://www.who.int/mediacentre/factsheets/fs211/en/ (Accessed: January 31, 2018).

^2^http://www.cytoscape.org/.

## Supplementary Material

Figure S1 Single-cell sorting approach for A2^+^M1_58_-specific CD8^+^ T cells from spleen/LN. Gating strategy for isolation of A2^+^M1_58_-specific CD8^+^ T from spleen and lymph node post magnetic enrichment. Single lymphocytes were gated on CD3^+^ cells and excluded for LIVE/DEAD^+^/CD14^+^/CD19^+^ cells (dump channel) before gating on A2^+^M1_58_-tetramer^+^CD8^+^ T cells. Frequencies shown are based on the parent gate, therefore, A2^+^M1_58_-tetramer^+^CD8^+^ T cell frequencies were calculated based on the total CD8^+^ T cell population.
