# Supplementary figures and images for: Single-Cell Approach to Influenza-Specific CD8+ T Cell Receptor Repertoires Across Different Age Groups, Tissues, and Following Influenza Virus Infection
Source: Front Immunol. 2018 Jun 27;9:1453. doi: 10.3389/fimmu.2018.01453 (PMC6030351; doi:10.3389/fimmu.2018.01453)

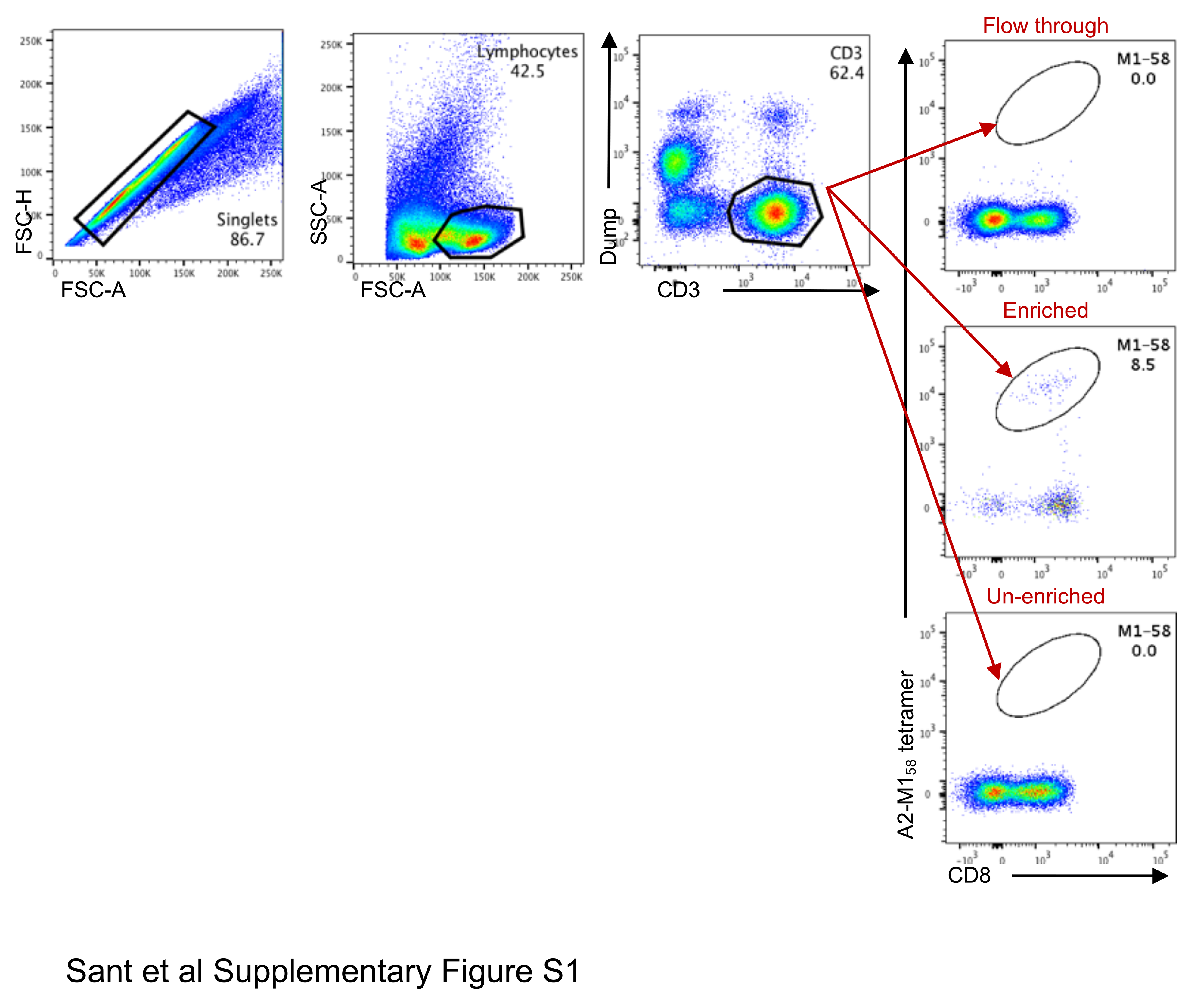

Supplement: Figure S1 — Single-cell sorting approach for A2+M158-specific CD8+ T cells from spleen/LN. Gating strategy for isolation of A2+M158-specific CD8+ T from spleen and lymph node post magnetic enrichment. Single lymphocytes were gated on CD3+ cells and excluded for LIVE/DEAD+/CD14+/CD19+ cells (dump channel) before gating on A2+M158-tetramer+CD8+ T cells. Frequencies shown are based on the parent gate, therefore, A2+M158-tetramer+CD8+ T cell frequencies were calculated based on the total CD8+ T cell population. [file Image_1.tif]
